# Translation and performance of the Finnish Diabetes Risk Score for detecting undiagnosed diabetes and dysglycaemia in the Indonesian population

**DOI:** 10.1371/journal.pone.0269853

**Published:** 2022-07-21

**Authors:** M. Rifqi Rokhman, Bustanul Arifin, Zulkarnain Zulkarnain, Satibi Satibi, Dyah Aryani Perwitasari, Cornelis Boersma, Maarten J. Postma, Jurjen van der Schans

**Affiliations:** 1 Department of Health Sciences, University of Groningen, University Medical Center Groningen, Groningen, The Netherlands; 2 Institute of Science in Healthy Ageing & healthcaRE (SHARE), University Medical Center Groningen, University of Groningen, Groningen, The Netherlands; 3 Faculty of Pharmacy, Universitas Gadjah Mada, Yogyakarta, Indonesia; 4 Faculty of Pharmacy, Universitas Hasanuddin, Makassar, Indonesia; 5 Faculty of Medicine, Public Health and Nursing, Universitas Gadjah Mada, Yogyakarta, Indonesia; 6 Unit of PharmacoTherapy, Epidemiology and Economics (PTE2), Department of Pharmacy, University of Groningen, Groningen, The Netherlands; 7 Faculty of Medicine, Universitas Syiah Kuala, Banda Aceh, Indonesia; 8 Thyroid Center, Zainoel Abidin Hospital, Banda Aceh, Indonesia; 9 Faculty of Pharmacy, Universitas Ahmad Dahlan, Yogyakarta, Indonesia; 10 Faculty of Management Sciences, Open University, Heerlen, The Netherlands; 11 Department of Pharmacology and Therapy, Faculty of Medicine, Universitas Airlangga, Surabaya, Indonesia; 12 Department of Economics, Econometrics and Finance, Faculty of Economics & Business, University of Groningen, Groningen, The Netherlands; Government College University Faisalabad, Pakistan, PAKISTAN

## Abstract

A diabetes risk score cannot directly be translated and applied in different populations, and its performance should be evaluated in the target population. This study aimed to translate the Finnish Diabetes Risk Score (FINDRISC) instrument and compare its performance with the modified version for detecting undiagnosed type 2 diabetes mellitus (T2DM) and dysglycaemia among the Indonesian adult population. Forward and backward translations were performed and followed by cultural adaptation. In total, 1,403 participants were recruited. The FINDRISC-Bahasa Indonesia (FINDRISC-BI) was scored according to the original FINDRISC instrument, while a Modified FINDRISC-BI was analyzed using a specific body mass index and waist circumference classification for Indonesians. The area under the receiver operating characteristic curve, sensitivity, specificity, and the optimal cut-offs of both instruments were estimated. The area under the receiver operating characteristic curve for detecting undiagnosed T2DM was 0.73 (0.67–0.78) for the FINDRISC-BI with an optimal cut-off score of ≥9 (sensitivity = 63.0%; specificity = 67.3%) and 0.72 (0.67–0.78) for the Modified FINDRISC-BI with an optimal cut-off score of ≥11 (sensitivity = 59.8%; specificity = 74.9%). The area under the receiver operating characteristic curve for detecting dysglycaemia was 0.72 (0.69–0.75) for the FINDRISC-BI instrument with an optimal cut-off score of ≥8 (sensitivity = 66.4%; specificity = 67.0%), and 0.72 (0.69–0.75) for the Modified FINDRISC-BI instrument with an optimal cut-off score ≥9 (sensitivity = 63.8%; specificity = 67.6%). The Indonesian version of the FINDRISC instrument has acceptable diagnostic accuracy for screening people with undiagnosed T2DM or dysglycaemia in Indonesia. Modifying the body mass index and waist circumference classifications in the Modified FINDRISC-BI results in a similar diagnostic accuracy; however, the Modified FINDRISC-BI has a higher optimal cut-off point than the FINDRISC-BI. People with an above optimal cut-off score are suggested to take a further blood glucose test.

## Introduction

The International Diabetes Federation estimates that the number of people living with diabetes was approximately 537 million in 2021, and this number is expected rise to almost 637 million at the end of 2030 [1]. Globally, the annual health expenditure to treat diabetes and its complications was at least USD 966 million. Indonesia ranks fifth among countries with the largest number of people with diabetes, and it was estimated 19.5 million people had diabetes in 2021 [1]. If the trend continues, the number is projected to reach 28.6 million in 2045. Worldwide, almost 50% of adults with diabetes are undiagnosed which represents the necessity of screening for diabetes [2].

Type 2 diabetes mellitus (T2DM) is a metabolic disorder due to progressive β-cell malfunction and insulin resistance characterized by multi-stimuli factors [3] and involving complex molecular mechanisms caused by mitochondrial, endothelial dysfunctions, and various inflammatory mechanisms [4, 5]. Prediabetes, as the early stage of diabetes, is characterized by a long asymptomatic stage with elevated blood glucose levels, leading to an increased risk of developing T2DM in the future [6]. Patients with abnormalities in their blood glucose levels including prediabetes and T2DM, defined as dysglycaemia [7], are generally associated with increased risk of death due to stroke, coronary heart disease, and peripheral vascular disease [8, 9].

Detection of dysglycaemia can substantially prevent or delay T2DM through intensive lifestyle modifications, long-term diet-based therapy [10] and/or pharmacological interventions [11–14], while early detection and treatment of people with T2DM are effective to reduce T2DM complications and the burden of disease [8, 15]. Many approaches have been developed and used to detect patients with T2DM including novel technologies based on metabolomics and genetic screening [16]. In the clinical setting, T2DM can be diagnosed based on plasma glucose criteria, either utilizing the fasting plasma glucose value, the oral glucose tolerance test, or the glycated haemoglobin (HbA1c) criteria [6]. However, these diagnostic methods are invasive and not suitable for screening the whole population since they are costly and time-consuming [17]. The identification of T2DM can be better addressed using a two-step approach, in which the use of diabetes risk scores as the first step is followed by a blood glucose test [17, 18]. Diabetes risk scores are expected to increase the cost-effectiveness of screening by significantly reducing the number of people who have to undergo invasive blood glucose tests [18, 19].

Several factors have been found to play important roles contributing to the risk of people for developing T2DM, such as obesity, poor diet, and low physical activity [20]. Based on these risk factors, a diabetes risk score was developed for detecting people with high risk of developing diabetes. The Finnish Diabetes Risk Score (FINDRISC) is a questionnaire used to identify individuals at risk of developing T2DM. In recent studies, the FINDRISC has also been evaluated as a tool to identify undiagnosed T2DM, dysglycaemia and metabolic syndrome [21–23]. Although the FINDRISC has been translated and validated in several languages [24–26], it was originally developed in Caucasian populations [27]. T2DM develops in East Asian populations at a lower mean body mass index (BMI) compared with those of other populations, for example, European descent [28]. In addition, the Asian populations have a higher body fat percentage at a lower BMI compared to Caucasians [28, 29]. Diabetes develops at a younger age in Asian patients, and is characterized by early β-cell dysfunction in the setting of insulin resistance [28]. Therefore, a diabetes risk score cannot be directly adopted and applied in different populations or ethnic groups, and its applicability should be evaluated in the target population, because modifications may be required [30, 31].

Modification of the FINDRISC is possible by revising the scoring system [32] or simplifying the instrument by selecting only the most relevant items based on logistic regression [22, 26]. To our knowledge, there are no existing translations of the FINDRISC nor the modifications of FINDRISC as diabetes risk scores for detecting diabetes in the Indonesian population. Therefore, the main aim of this study was to translate the FINDRISC instrument and compare its performance with the modified version for detecting undiagnosed T2DM and dysglycaemia among the Indonesian adult population. The second aim was to determine the association between the FINDRISC components with the detection of T2DM and dysglycaemia.

## Material and methods

### Study setting

The translation, cultural adaptation, and testing the performance of the FINDRISC instrument were conducted in two regions in Java Island (Yogyakarta Province and Malang, East Java Province) and a regency in Sulawesi Island (Banggai Laut, Central Sulawesi Province). Of the 34 provinces in Indonesia, there are 12 provinces where the prevalence of patients with T2DM is higher than the national average prevalence level, including Yogyakarta, East Java, and Central Sulawesi [33]. We conducted the translation phase in the last week of April 2019, the cultural adaptation phase in May 2019, and the performance test of FINDRISC from June to November 2019.

### Participants

Our target population were community members and government employees. We contacted them by asking permission from the head of the institution or community leader followed by an explanation of the objectives, procedures and research ethics. Participants were people who had never been diagnosed with diabetes mellitus (types 1 and 2) and aged minimum 18 years. After receiving information about this study, all prospective participants should provide written informed consent in order to participate in this study. A day before, we reminded all potential participants to fast for 8 hours and only allowed to drink plain water before we assessed their fasting blood glucose (FBG). Exclusion criteria were participants who were using drugs that could affect blood glucose levels (i.e., thiazides, beta-blockers, and steroids) or participants with diseases or clinical conditions that affected blood glucose levels (i.e., anorexia nervosa, hepatitis and pancreatic tumors). Participant selection happened in the same manner in each separate phase.

In the performance test, the minimum number of participants was determined based on the Burderer’s formula for sensitivity in diagnostic health studies and incorporated the prevalence of the disease in the formula 1 [34, 35].


$$\text{Minimum}\;\text{participants}\;\text{based}\;\text{on}\;\text{sensitivity}=\frac{Z_{\propto /2}^{2}\;\text{x}\;\hat{\text{Sensitivity}}\;\text{x}\;(1-\text{Sensitivity})}{{\text{W}}^{2}\;\text{x}\;\text{Prevalence}}$$ (1)

For the level of confidence of 95% (α = 0.05), the value of *Z*_∝/2_ is 1.96. Based on a previous study, the sensitivity of FINDRISC for undiagnosed diabetes based on FBG ($\hat{\text{Sensitivity}}$) was 0.81 [36]. The maximum acceptable width of the 95% confidence interval (W) was set to 10%. The prevalence of diabetes in Indonesia is 6.2% [2]. Therefore, a minimum total of 954 participants was required for the performance test.

### Instrument

The research instruments consisted of a socio-demographic form and the Bahasa Indonesia version of the original FINDRISC. Socio-demographic data included age, sex, and education level. The original FINDRISC instrument consists of 8 items concerning risk-factors related to T2DM, namely age, BMI, waist circumference, daily physical activity, consumption of vegetables and fruit, consumption of hypertension drugs, history of high blood glucose and family history of diabetes. Each item consists of several answer choice scales [27].

### Study procedure and data collection

#### Translation and cultural adaptation

The translation stage consisted of both a forward and backward translation [37, 38]. In the forward translation, the original FINDRISC instrument was translated from English to Bahasa Indonesia by two Indonesian professional translators working independently. Then, in the backward translation, the translated document was sent to two native speakers of English (also fluent in Bahasa Indonesia) to translate the instrument from Bahasa Indonesia to English. The main purpose of backward translation is to ensure that the translation in the forward translation phase was correct and accurate [39]. The final document of each step was reviewed and discussed by the Indonesian research team. If there were differences of opinion including with and between the translators, the final version was decided upon by consensus.

The initial FINDRISC in Bahasa Indonesia was tested on 10 participants in Sulawesi and 10 participants in Java [39]. The participants were asked whether the items were understandable to them and what their opinion was on each instrument item. At this stage, we replaced the word ‘berries’ with ‘fruits’ because berries are not a popular product in Indonesia, and this might not be understood by Indonesian people. The final product from this phase was the result of an agreement of the Indonesian research team. This version was labelled ‘FINDRISC-Bahasa Indonesia (FINDRISC-BI)’.

#### Data collection for performance test

The FINDRISC-BI was distributed among study participants in this phase. The study instrument was filled in by the participants themselves followed by the measurement of participants’ FBG, height, weight and waist circumference.

FBG was measured by a physician or nurse using a finger-stick blood glucose test (Easy Touch®GCU), while the measurements of weight, height and waist circumference were carried out by trained research assistants. Participants’ weight was measured in kilograms using electronic scales with a precision of 0.1 kg, while their height was measured in centimeters with a precision of 0.1 centimeters. When weighing, we asked participants to take off their footwear and only wear light clothing. BMI was measured based on the weight and height of each participant. Waist circumference was measured using a non-stretchy tape with a precision of 0.1 centimeters.

### Definition of undiagnosed T2DM and dysglycaemia

The Indonesian Society of Endocrinology divides FBG into 3 categories, namely: normal (FBG <100mg/dL), prediabetes (100-125mg/dL) and T2DM (≥126mg/dL) [40]. In this study, participants with abnormal FBG were defined as dysglycaemia (prediabetes and T2DM combined) while participants with FBG higher than 126mg/dL were labelled as undiagnosed T2DM.

### Definition of the FINDRISC-BI and Modified FINDRISC-BI

The FINDRISC instrument uses BMI and waist circumference based on the Caucasian ethnic group [27]. In the FINDRISC, BMI was classified into 3 groups (0 points for BMI <25kg/m^2^, 1 point for BMI 25-30kg/m^2^, and 3 points for BMI >30kg/m^2^), while waist circumference was categorised into 3 groups (for males 0 points <94cm, 3 points 94–102, 4 points >102cm; for female 0 points <80cm, 3 points 80–88, 4 points >88cm). Besides scoring the FINDRISC-BI based on the scoring of the original FINDRISC, we also classified the BMI and waist circumference item of the FINDRISC using the classification from the Indonesian Health Ministry. In this Modified FINDRISC-BI, BMI was classified into 3 groups (0 points for BMI <25kg/m^2^, 1 point for BMI 25-27kg/m^2^, and 3 points for >27kg/m^2^), with only 2 groups for waist circumference classification (for males 0 points <90cm, 4 points ≥90cm; for females 0 points <80cm, 4 points ≥80cm). The comparison of scoring between the FINDRISC-BI and Modified FINDRISC-BI can be seen in S1 Table, while S2 Table presents the Modified FINSRISC-BI instrument.

### Data analysis

Participants’ characteristics were described as frequencies and percentages for categorical data, while means ± standard deviations (SD) for continuous data were presented. Comparisons between the groups were analyzed with Chi-square tests for categorical data and unpaired t-tests for continuous data.

The accuracy of the FINDRISC-BI was analyzed using the area under the receiver operating characteristic (ROC) curve. The sensitivity, specificity, false positive, false negative, positive predictive value, and negative predictive value were calculated for both the FINDRISC-BI and Modified FINDRISC-BI. The optimal cut-offs for detecting undiagnosed T2DM and dysglycaemia were calculated by estimating the point with the shortest distance to (0,1) in the ROC curve that maximizes both the sensitivity and specificity. The distance for each observed cut-off was determined as the square root of [(1-Sensitivity)^2^ + (1-Specificity)^2^] [21, 41].

The associations of the FINDRISC-BI and Modified FINDRISC-BI components with undiagnosed T2DM or dysglycaemia were analyzed using multivariate logistic regression analysis. Independent variables were all components of the FINDRISC-BI or Modified FINDRISC-BI, while undiagnosed T2DM and dysglycaemia were defined as dependent variables. All statistical analyses were performed using the Statistical Package for Social Sciences (SPSS Inc., Chicago, IL, USA), version 26.0. The level of statistical significance was set at *p* < .05.

### Ethics statement

The study was approved by the Ethics Committee of the Faculty of Dentistry of Universitas Gadjah Mada, Yogyakarta, Indonesia in document number 0095/KKEP/FKG-UGM/ES/2019 on April 25, 2019. Permission to develop and publish an Indonesian version of the FINDRISC instrument was obtained from Jaana Lindström, MSC and Jaakko Tuomilehto, MD, PHD, and the American Diabetes Association as the copyright holder.

## Results

No significant changes were made from the original FINDRISC English version in the translation process. In the cultural adaptation step, twenty participants participated. Some of the participants questioned item number 4 ‘do you usually have at least 30 minutes of daily physical activity at work or during leisure time (including normal daily activity)?’. They asked for further explanation regarding ‘daily physical activity’ and whether this referred to ‘minimal 30 minutes exercise in a day’ or ‘all kind of activities for 30 minutes in a day’?. To address this issue, this specific item was discussed with the original author, who stated that ‘daily physical activity’ referred to ‘all kind of activities for 30 minutes in a day’.

Furthermore, some participants also had difficulty in differentiating between answer choice “Yes: grandparent, aunt, uncle, or first cousin (but no own parent, brother, sister, or child) (3 points)” and “Yes: parent, brother, sister, or own child (5 points)” of the last question of the FINDRISC. Therefore, we agreed to change the order of answer choices to “No (0 points)”; “Yes: parent, brother, sister, or own child (5 points)”; and “Yes: grandparent, aunt, uncle, or first cousin (but no own parent, brother, sister, or child) (3 points). This change aimed to facilitate communication between researchers and participants when explaining this item. Participants will more easily remember the history of their immediate family illness (parents and siblings) compared to the history of their grandparents.

### Characteristics of study participants

Table 1 shows the descriptive characteristics of 1,403 participants. The majority of the participants were women (61.4%), had a mean age of 41.9 years old (SD = 15.4), education of diploma or higher (51.2%), average waist diameter of 86.0 cm (SD = 12.1), BMI of 24.5 (SD = 4.5), and FBG of 97.5 mg/dL (SD = 29.8). The number of participants with dysglycaemia was 426 (30.4%), and 92 participants (6.6%) were labelled as undiagnosed T2DM. In general, participants with dysglycaemia had a higher risk factor profile in terms of their age, waist circumference, BMI, history of antihypertensive medication use, history of high blood glucose and having a first-degree relative with diabetes compared to those without dysglycaemia. Similarly, participants with undiagnosed T2DM had a higher risk factor profile compared to participants without undiagnosed T2DM.

**Table 1:** Descriptive characteristics of study participants (n = 1,403).

	Undiagnosed T2DM		*p*-value	Dysglycaemia		*p*-value	Total
	No (n = 1,311)	Yes (n = 92)		No (n = 977)	Yes (n = 426)		
Study sites, n (%)							
Java	717 (54.7)	60 (65.2)	0.050	525 (53.7)	252 (59.2)	0.060	777 (55.4)
Sulawesi	594 (45.3)	32 (34.8)		452 (46.3)	174 (40.8)		626 (44.6)
Sex (female), n (%)	801 (61.1)	60 (65.2)	0.433	610 (62.4)	251 (58.9)	0.214	861 (61.4)
Education (diploma or higher), n (%)	693 (52.9)	25 (27.2)	<0.001	518 (53.0)	200 (46.9)	0.036	718 (51.2)
Age (years), mean (SD)	41.0 (15.2)	54.3 (12.8)	<0.001	39.2 (14.8)	48.0 (15.2)	<0.001	41.9 (15.4)
Body mass index (kg/m^2^), mean (SD)	24.5 (4.4)	25.4 (4.8)	0.052	24.0 (4.2)	25.9 (4.7)	<0.001	24.5 (4.5)
Waist circumference (cm), mean (SD)	85.8 (12.0)	89.4 (12.3)	0.006	84.3 (11.7)	89.9 (12.1)	<0.001	86.0 (12.1)
Physical activity (30 minutes/day) (yes), n (%)	989 (75.4)	63 (68.5)	0.136	741 (75.8)	311 (73.0)	0.259	1052 (75.0)
Consumption of vegetables and fruit (yes), n (%)	554 (42.3)	37 (40.2)	0.702	402 (41.1)	189 (44.4)	0.261	591 (42.1)
Antihypertensive medication (yes), n (%)	182 (13.9)	30 (32.6)	<0.001	98 (10.0)	114 (26.8)	<0.001	212 (15.1)
History of high blood glucose (yes), n (%)	117 (8.9)	36 (39.1)	<0.001	61 (6.2)	92 (21.6)	<0.001	153 (10.9)
Family members with diabetes (yes), n (%)							
Non-first-degree relative	147 (11.2)	3 (3.3)	0.002	106 (10.8)	44 (10.3)	<0.001	150 (10.7)
First-degree relative	295 (22.5)	33 (35.9)		180 (18.4)	148 (34.7)		328 (23.4)
Fasting blood glucose (mg/dL), mean (SD)	91.7 (12.5)	180.7 (62.6)	<0.001	86.2 (8.8)	123.5 (42.1)	<0.001	97.5 (29.8)
FINDRISC-BI score, mean (SD)	6.7 (4.5)	11.2 (5.7)	<0.001	5.9 (4.1)	9.7 (5.0)	<0.001	7.0 (4.7)
Modified FINDRISC-BI score, mean (SD)	7.5 (4.8)	12.0 (5.8)	<0.001	6.6 (4.5)	10.5 (5.2)	<0.001	7.8 (5.0)

T2DM: type 2 diabetes mellitus; FINDRISC-BI: Finnish Diabetes Risk Score-Bahasa Indonesia; SD: standard deviation.

### Diagnostic accuracy

**Fig 1:**
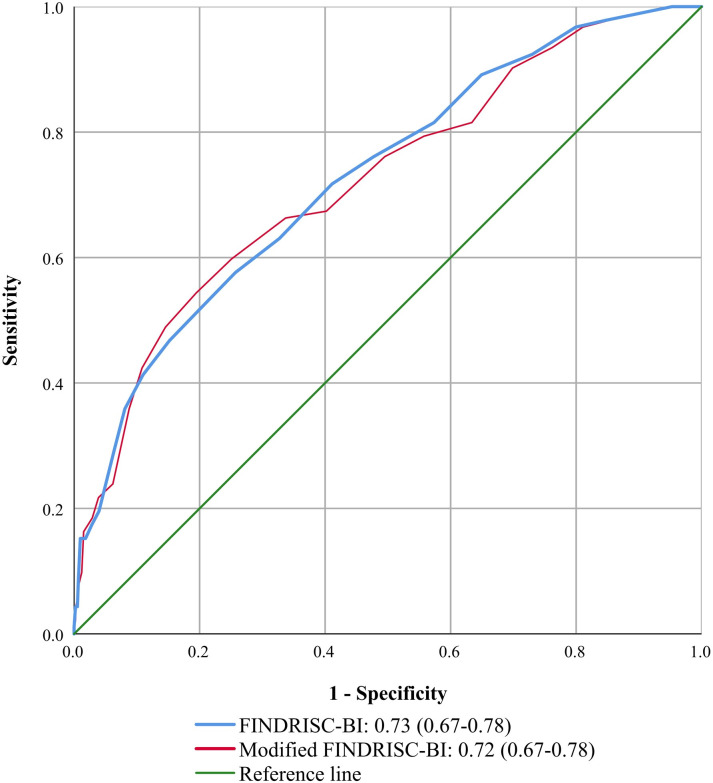
ROC curves for undiagnosed T2DM. FINDRISC-BI: Finnish Diabetes Risk Score-Bahasa Indonesia; T2DM: type 2 diabetes mellitus; ROC curve: receiver operating characteristic curve.

**Fig 2:**
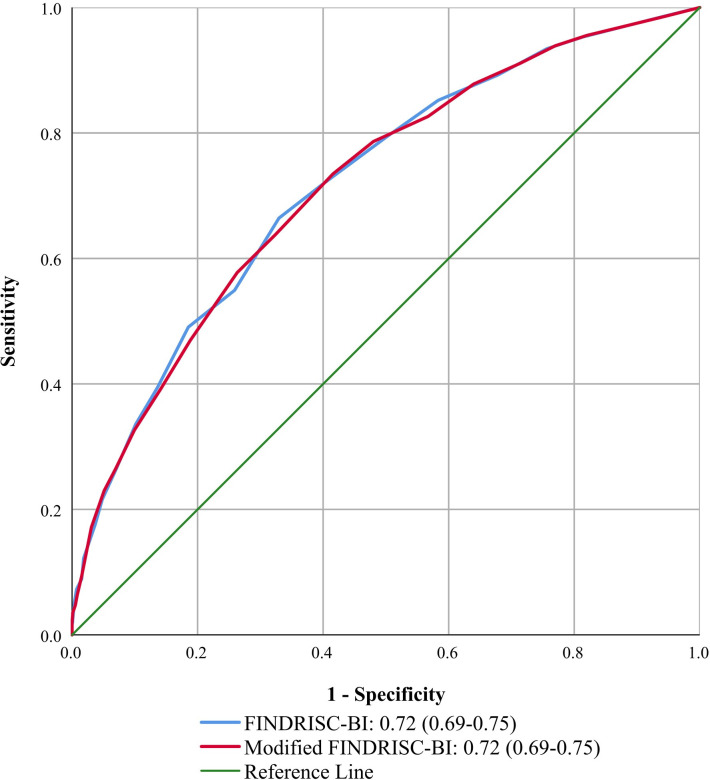
ROC curves for dysglycaemia. FINDRISC-BI: Finnish Diabetes Risk Score-Bahasa Indonesia; T2DM: type 2 diabetes mellitus; ROC curve: receiver operating characteristic curve.

The diagnostic accuracy between the FINDRISC-BI and Modified FINDRSC-BI was similar (Figs 1 and 2). The area under the ROC curve for detecting undiagnosed T2DM was 0.73 (0.67–0.78) for the FINDRISC-BI with an optimal cut-off score of ≥9 (sensitivity = 63.0%; specificity = 67.3%) and 0.72 (0.67–0.78) for the Modified FINDRISC-BI with an optimal cut-off score of ≥11 (sensitivity = 59.8%; specificity = 74.9%) (Table 2). The area under the ROC curve for detecting dysglycaemia was 0.72 (0.69–0.75) for the FINDRISC-BI with an optimal cut-off score of ≥8 (sensitivity = 66.4%; specificity = 67.0%), and 0.72 (0.69–0.75) for the Modified FINDRISC-BI with an optimal cut-off score of ≥9 (sensitivity = 63.8%; specificity = 67.6%). S3 Table provides information about the characteristics of the FINDRISC-BI and Modified FINDRISC-BI using different cut-offs for detecting dysglycaemia and undiagnosed T2DM.

**Table 2:** Characteristics of FINDRISC-BI and Modified FINDRISC-BI using the optimal cut-off scores to detect undiagnosed T2DM and dysglycaemia.

	Undiagnosed T2DM		Dysglycaemia	
	FINDRISC-BI	Modified FINDRISC-BI	FINDRISC-BI	Modified FINDRISC-BI
Cut-off point	9	11	8	9
Sensitivity, %	63.0	59.8	66.4	63.8
Specificity, %	67.3	74.9	67.0	67.6
False positive, %	32.7	25.1	33.0	32.4
False negative, %	37.0	40.2	33.6	36.2
Positive predictive value, %	11.9	14.3	46.8	46.2
Negative predictive value, %	96.3	96.4	82.1	81.1

FINDRISC-BI: Finnish Diabetes Risk Score-Bahasa Indonesia; T2DM: type 2 diabetes mellitus.

### FINDRISC components and undiagnosed T2DM or dysglycaemia

Table 3 describes the associations of the FINDRISC-BI and Modified FINDRISC-BI with undiagnosed T2DM or dysglycaemia from the multivariate logistic regression analysis. For the FINDRISC-BI, components that were associated with undiagnosed T2DM were age, waist circumference, consumption of vegetables and fruit and history of high blood glucose, while in the Modified FINDRISC-BI only age and history of high blood glucose were associated with undiagnosed T2DM. For both the FINDRISC-BI and Modified FINDRISC-BI, components that were associated with dysglycaemia were age, BMI, taking antihypertensive medication, history of high blood glucose and family members with diabetes mellitus. One component, namely physical activity, was not significant in both of the models for detecting undiagnosed T2DM and dysglycaemia.

**Table 3:** Multivariate logistic regression model of association between the FINDRISC-BI and Modified FINDRISC-BI, and undiagnosed T2DM or dysglycaemia in total participants (n = 1,403).

	Undiagnosed T2DM				Dysglycaemia			
	FINDRISC-BI		Modified FINDRISC-BI		FINDRISC-BI		Modified FINDRISC-BI	
	OR	(95% CI)	OR	(95% CI)	OR	(95% CI)	OR	(95% CI)
Age								
<45 years	1.00		1.00		1.00		1.00	
45–54 years	3.64	(1.92–6.89)***	3.78	(2.00–7.14)***	1.92	(1.37–2.71)***	1.89	(1.34–2.67)***
55–64 years	3.62	(1.73–7.56)***	3.94	(1.91–8.15)***	2.81	(1.88–4.2)***	2.84	(1.91–4.23)***
>64 years	7.00	(3.68–13.32)***	7.21	(3.79–13.70)***	3.85	(2.58–5.73)***	3.84	(2.58–5.71)***
Body mass index^a^								
<25 kg/m^2^	1.00		1.00		1.00		1.00	
25–30 kg/m^2^	0.94	(0.55–1.62)	1.01	(0.53–1.93)	1.60	(1.18–2.17)**	1.52	(1.06–2.19)*
>30 kg/m^2^	0.70	(0.31–1.62)	0.93	(0.52–1.68)	2.17	(1.35–3.47)**	1.94	(1.39–2.71)***
Waist circumference^b^								
Female:<80 cm, male:<94 cm	1.00		1.00		1.00		1.00	
Female:80–88 cm, male:94–102 cm	1.31	(0.70–2.44)	1.48	(0.84–2.62)	1.16	(0.84–1.62)	1.32	(0.97–1.81)
Female:>88 cm, male:>102 cm	1.89	(1.00–3.58)*			1.41	(0.98–2.04)		
Physical activity (30 minutes/day)								
Yes	1.00		1.00		1.00		1.00	
No	1.04	(0.81–1.35)	1.05	(0.81–1.35)	1.05	(0.91–1.21)	1.05	(0.91–1.21)
Consumption of vegetables and fruit								
Every day	1.00		1.00		1.00		1.00	
Not every day	1.63	(1.00–2.64)*	1.60	(0.99–2.60)	1.11	(0.86–1.45)	1.10	(0.85–1.43)
Antihypertensive medication								
No	1.00		1.00		1.00		1.00	
Yes	1.05	(0.80–1.39)	1.05	(0.79–1.39)	1.30	(1.09–1.55)**	1.31	(1.10–1.56)**
History of high blood glucose								
No	1.00		1.00		1.00		1.00	
Yes	1.39	(1.25–1.56)***	1.40	(1.25–1.56)***	1.19	(1.10–1.29)***	1.19	(1.10–1.29)***
Family members with diabetes								
No	1.00		1.00		1.00		1.00	
Grandparent, aunt, uncle or first cousin	0.48	(0.14–1.67)	0.48	(0.14–1.68)	1.65	(1.07–2.53)*	1.65	(1.08–2.53)*
Parent, brother, sister or own child	1.28	(0.77–2.15)	1.31	(0.79–2.19)	2.22	(1.66–2.98)***	2.24	(1.67–3.00)***

FINDRISC-BI: Finnish Diabetes Risk Score-Bahasa Indonesia; T2DM: type 2 diabetes mellitus; OR: odds ratio; 95% CI: 95% confidence interval.

^a^Body mass index in the Modified FINDRISC-BI was classified into <25 kg/m^2^, 25–27 kg/m^2^, and >27 kg/m^2^.

^b^Waist circumference in the Modified FINDRISC-BI only consists of two groups (female:<80 cm or male:<90 cm and female:≥ 80 cm or male:≥90 cm).

*p-value < 0.05;

**p-value < 0.01;

***p-value < 0.001.

## Discussion

Our study shows that both the FINDRISC-BI and Modified FINDRISC-BI instruments have acceptable diagnostic accuracy in the Indonesian population to detect people with T2DM or dysglycaemia. The area under the ROC curve of both instruments is in the range of 0.7–0.8, which is considered as acceptable for an instrument to discriminate between people with and without undiagnosed T2DM or with and without dysglycaemia [42]. Our study showed a higher diagnostic accuracy compared to two previous studies of diabetes risk assessment tools in Indonesia with an area under the ROC of 0.65 and 0.64 for detecting prediabetes and T2DM patients respectively [43, 44]. The finding is also comparable with other studies in the Asian setting using the FINDRISC instrument, reporting an area under the ROC of 0.78 in the rural population of China [45], 0.74 in the Philippines [46], 0.77 in India [47], and 0.76 (undiagnosed T2DM) and 0.79 (dysglycaemia) in Malaysia [32].

Daily physical activity was not significantly associated with both undiagnosed T2DM and dysglycaemia. In the development of the original FINDRISC, this variable did not significantly increase the predictive power but was still included among the modifiable variables [27]. In our study, the absence of an association for physical activity could also be related to the fact that our participants potentially incorrectly responded to this question and needed a more detailed question. Two meta-analysis studies showed that the intensity of physical activity (moderate to high) was associated with reductions in HbA1C [48, 49], which could indicate a stricter definition of the daily physical activity item. Therefore, future studies in Indonesia should demonstrate whether the incorporation and clearer definitions of the minimum intensity of physical activity add to the distinctive power of the FINDRISC instrument.

The diagnostic accuracy of both the FINDRISC-BI and Modified FINDRISC-BI for screening undiagnosed T2DM and dysglycaemia is similar. Modifying the BMI and waist circumference classifications did not significantly impact the area under the ROC curve. This finding was similar to two other studies that reported that adjusting the BMI and waist circumference according to the specific Asia-Pacific population did not show significant differences between the FINDRISC and the modified version in the Philippines and Malaysia [32, 46].

Although the performance of both instruments is similar, we suggest the use of the Modified FINDRISC-BI over the FINDRISC-BI, since lower obesity cut-off points are needed in South Asians to detect an equivalent level of dysglycaemia as observed in Caucasian Europeans for which the FINDRISC was first developed [50, 51]. The Modified FINDRISC-BI with a cut-off point of 11 had a slightly lower sensitivity than FINDRISC-BI with a cut-off point of 9 in detecting undiagnosed T2DM, but the Modified FINDRISC-BI had higher specificity, positive predictive value, and negative predictive value. As a result, the Modified FINDRISC-BI offered a lower proportion of the participants with the FINDRISC score above the cut-off point or lower proportion of people that should undergo further diagnostic testing. For practical reasons, the classification of BMI and waist circumference in the Modified FINDRIC-BI will ease the education or counselling to the community since the classifications are similar to the health campaigns promoted by the Indonesian Ministry of Health.

The findings of our study will guide future studies in adopting and evaluating the use of diabetes risk scores in the Indonesian health care system. The Modified FINDRISC-BI can be used in several ways to support the national strategy for reducing the incidence of diabetes. This instrument can be incorporated with the Mobile *Jaminan Kesehatan Nasional*, a mobile screening application launched by the Indonesian national health insurance system. This instrument can also be used by healthcare professionals to screen high-risk people at low costs. Further diagnostic testing, such as oral glucose tolerance tests, should be conducted for people with a Modified FINDRISC-BI score of 11 or higher for detecting undiagnosed T2DM, while people with a Modified FINDRISC-BI score of 9 or higher should be tested for possible abnormality of their blood glucose level. Incorporating a risk score with a diagnostic test, which could enhance the cost-effectiveness of the population-based screening, is considered more efficient than screening using the diagnostic test alone [52]. Therefore, it is also important for future studies to evaluate the cost-effectiveness of screening for diabetes using a combination of FINDRISC and blood glucose testing.

The remaining pivotal issue for using the FINDRISC score is the impact of the false-positive and false-negative groups. The number of people in the false-positive group can be significantly minimized through further blood glucose tests as the next step of diagnosis. However, for people in the false-negative group, there are potential risks of adverse outcomes in individuals with undiagnosed diabetes who would not be detected by a risk score approach [53]. This can be minimized by conducting repeated testing at a regular interval [54].

The prevalence of undiagnosed T2DM in this study was 6.6%, which is close to the estimation of the prevalence of diabetes in Indonesia estimated by the International Diabetes Federation in 2019 (6.2%) and by the National Baseline Health Research in 2018 (8.5%) [2, 33]. Our findings also demonstrated that the prevalence of dysglycaemia in the participants was high (30.4%). This is slightly below the estimated prevalence of dysglycaemia (impaired fasting glucose and diabetes) in the Indonesian adult population (34.8%) in 2018 [33]. The finding suggests more intensive screening is needed to detect people with dysglycaemia.

To our knowledge, this is the first study to evaluate the performance of the FINDRISC instrument in the Indonesian population. The strength of this research is that participants were taken from two big islands with the highest prevalence of patients with T2DM in Indonesia. Sampling was done so that our study population was representative for the whole of Indonesia, with Java representing the population of the western part of Indonesia and Sulawesi representing the central and eastern part of Indonesia. This study also covered a broad range of participants in terms of age and education.

The results should be interpreted in the light of the study’s limitations. In this research, the parameter to define a participant having prediabetes or T2DM was solely based on the participant’s capillary FBG level using a finger-stick blood glucose test. However, this method was the only reliable method available since we collected data not only in participants from rural areas but also those who lived in very remote areas. Although the performance of a FBG test is less sensitive compared to an oral glucose tolerance test [55], the Indonesian Society of Endocrinology and the American Diabetes Association still include this method as a valid tool for the diagnosis of diabetes mellitus [6, 40]. Another limitation is the use of a cross-sectional study design, which means that the study is limited to detecting people with existing T2DM or dysglycaemia.

## Conclusions

The Indonesian version of the FINDRISC instrument has acceptable diagnostic accuracy for screening people with undiagnosed T2DM or dysglycaemia in Indonesia. Modifying the BMI and waist circumference classifications in the Modified FINDRISC-BI results in a similar diagnostic accuracy; however, the Modified FINDRISC-BI has a higher optimal cut-off point than the FINDRISC-BI. People with an above optimal cut-off score are suggested to take a further blood glucose test. The associations between the FINDRISC-BI and Modified FINDRISC-BI components with undiagnosed T2DM or dysglycaemia demonstrate that daily physical activity was not significantly associated with both undiagnosed T2DM and dysglycaemia.

## Supporting information

S1 Dataset (XLSX)

S1 Table Scoring of FINDRISC-BI and Modified FINDRISC-BI. (DOCX)

S2 Table The Modified FINDRISC-Bahasa Indonesia instrument. (DOCX)

S3 Table Characteristics of FINDRISC-BI and Modified FINDRISC-BI using different cut-offs for detecting undiagnosed T2DM and dysglycaemia. (DOCX)

S1 File Research protocol. (DOCX)
