# Supplementary material for: Translation and performance of the Finnish Diabetes Risk Score for detecting undiagnosed diabetes and dysglycaemia in the Indonesian population
Source: PLoS One. 2022 Jul 21;17(7):e0269853. doi: 10.1371/journal.pone.0269853 (PMC9302803; doi:10.1371/journal.pone.0269853)
Supplement: S1 Table — (DOCX) [file pone.0269853.s002.docx]

**S1 Table.** **Scoring of FINDRISC-BI and Modified FINDRISC-BI.**

|  | **FINDRISC-BI*** | **Modified FINDRISC-BI*** |
| --- | --- | --- |
| Age |  |  |
| <45 years | 0 points | 0 points |
| 45-54 years | 2 points | 2 points |
| 55-64 years | 3 points | 3 points |
| >64 years | 4 points | 4 points |
| Body Mass Index |  |  |
| <25 kg/m^2^ | 0 points | 0 points (<25 kg/m^2^) |
| 25-30 kg/m^2^ | 1 point | 1 point (25-27 kg/m^2^) |
| >30 kg/m^2^ | 3 points | 3 points (>27 kg/m^2^) |
| Waist circumference |  |  |
| Female <80 cm, Male <94 cm | 0 points | 0 points (Female <80 cm, Male <90 cm) |
| Female 80-88 cm, Male 94-102 cm | 3 points | - |
| Female >88 cm, Male >102 cm | 4 points | 4 points (Female >80 cm, Male >90 cm) |
| Physical activity (30 minutes/day) |  |  |
| Yes | 0 points | 0 points |
| No | 2 points | 2 points |
| Fruit and vegetable consumption |  |  |
| Every day | 0 points | 0 points |
| Not every day | 1 point | 1 point |
| Antihypertensive medication |  |  |
| No | 0 points | 0 points |
| Yes | 2 points | 2 points |
| History of high blood glucose |  |  |
| No | 0 points | 0 points |
| Yes | 5 points | 5 points |
| Family members with diabetes |  |  |
| No | 0 points | 0 points |
| Grandparent, aunt, uncle or first cousin | 3 points | 3 points |
| Parent, brother, sister or own child | 5 points | 5 points |

The difference between the FINDRISC-BI and Modified FINDRISC-BI is based on the scores and the body mass index and waist circumference categories.

*Adapted with permission from Jaana Lindström, MSC and Jaakko Tuomilehto, MD, PHD; The Diabetes Risk Score: A practical tool to predict type 2 diabetes risk. *Diabetes Care* 2003;26(3):725–731, https://doi.org/10.2337/diacare.26.3.725. Copyright 2003 by the American Diabetes Association.
