# Supplementary material for: Translation and performance of the Finnish Diabetes Risk Score for detecting undiagnosed diabetes and dysglycaemia in the Indonesian population
Source: PLoS One. 2022 Jul 21;17(7):e0269853. doi: 10.1371/journal.pone.0269853 (PMC9302803; doi:10.1371/journal.pone.0269853)
Supplement: S2 Table — (DOCX) [file pone.0269853.s003.docx]

**S2 Table.** **The Modified FINDRISC-Bahasa Indonesia instrument*.**

| **No** | **Items** | **Pilihan** | **Score** |
| --- | --- | --- | --- |
| 1 | Usia | Di bawah 45 tahun | 0 |
|  |  | 45-54 tahun | 2 |
|  |  | 55-64 tahun | 3 |
|  |  | Di atas 64 tahun | 4 |
| 2 | Indeks massa tubuh | Kurang dari 25 kg/m^2^ | 0 |
|  |  | 25-27 kg/m^2^ | 1 |
|  |  | Lebih dari 27 kg/m^2^ | 3 |
| 3 | Lingkar pinggang diukur dari bawah tulang rusuk (biasanya sejajar dengan pusar) | Wanita <80 cm, Pria <90 cm | 0 |
|  |  | Wanita >80 cm, Pria >90 cm | 4 |
| 4 | Apakah Anda rutin melakukan aktivitas fisik minimal 30 menit, baik saat anda sedang bekerja maupun saat anda sedang luang (termasuk aktivitas sehari-hari)? | Ya | 0 |
|  |  | Tidak | 2 |
| 5 | Seberapa sering Anda konsumsi sayur, buah? | Setiap hari | 0 |
|  |  | Tidak setiap hari | 1 |
| 6 | Apakah Anda pernah konsumsi obat hipertensi secara teratur (minimal selama 1 bulan)? | Tidak | 0 |
|  |  | Ya | 2 |
| 7 | Apakah Anda pernah mendapatkan bahwa hasil pemeriksaan gula darah anda tinggi? (contohnya saat tes kesehatan untuk suatu keperluan; saat sakit tertentu; atau selama kehamilan)? | Tidak | 0 |
|  |  | Ya | 5 |
| 8 | Apakah anggota keluarga kandung Anda atau kerabat lainnya pernah didiagnosa mengidap diabetes (tipe 1 atau tipe 2)? | Tidak | 0 |
|  |  | Ya: orang tua, kakak atau adik kandung | 5 |
|  |  | Ya: kakek dan/atau nenek, bibi, paman, atau sepupu, bibi, paman atau sepupu pertama (tidak sekandung/ bukan berasal dari orang tua yang sama) | 3 |

| Skor Total Risiko | **…** |
| --- | --- |
|  |  |
| **Skor** | **Risiko perkembangan diabetes tipe 2 selama 10 tahun** |
| Kurang dari 7 | ***Rendah:*** diperkirakan 1 dari 100 mengembangkan penyakit |
| 7–11 | ***Cukup Rendah:*** diperkirakan 1 dari 25 mengembangkan penyakit |
| 12–14 | ***Sedang:*** diperkirakan 1 dari 6 mengembangkan penyakit |
| 15–20 | ***Tinggi:*** diperkirakan 1 dari 3 mengembangkan penyakit |
| Lebih dari 20 | ***Sangat tinggi:*** diperkirakan 1 dari 2 mengembangkan penyakit |

*Adapted with permission from Jaana Lindström, MSC and Jaakko Tuomilehto, MD, PHD; The Diabetes Risk Score: A practical tool to predict type 2 diabetes risk. *Diabetes Care* 2003;26(3):725–731, https://doi.org/10.2337/diacare.26.3.725. Copyright 2003 by the American Diabetes Association.
