# Supplementary material for: Translation and performance of the Finnish Diabetes Risk Score for detecting undiagnosed diabetes and dysglycaemia in the Indonesian population
Source: PLoS One. 2022 Jul 21;17(7):e0269853. doi: 10.1371/journal.pone.0269853 (PMC9302803; doi:10.1371/journal.pone.0269853)
Supplement: S3 Table — (DOCX) [file pone.0269853.s004.docx]

**S3 Table.** **Characteristics of FINDRISC-BI and Modified FINDRISC-BI using different cut-offs for detecting undiagnosed T2DM and dysglycaemia.**

| **Cut-off** | **Sen (%)** | **Spe (%)** | **FP (%)** | **FN (%)** | **PPV (%)** | **NPV (%)** | **√[(1-Sen)^2^ + (1-Spe)^2^]** | **n (%)*** |
| --- | --- | --- | --- | --- | --- | --- | --- | --- |
| **Undiagnosed T2DM** | | | | | | | | |
| **FINDRISC-BI** | | | | | | | | |
| 7 | 76.1 | 52.2 | 47.8 | 23.9 | 10.0 | 96.9 | 0.534 | 697 (49.7) |
| 8 | 71.7 | 58.9 | 41.1 | 28.3 | 10.9 | 96.7 | 0.499 | 605 (43.1) |
| **9** | **63.0** | **67.3** | **32.7** | **37.0** | **11.9** | **96.3** | **0.494** | **487 (34.7)** |
| 10 | 57.6 | 74.3 | 25.7 | 42.4 | 13.6 | 96.2 | 0.496 | 390 (27.8) |
| 11 | 51.1 | 80.6 | 19.4 | 48.9 | 15.6 | 95.9 | 0.526 | 301 (21.5) |
| **Modified FINDRISC-BI** | | | | | | | | |
| 9 | 67.4 | 59.8 | 40.2 | 32.6 | 10.5 | 96.3 | 0.518 | 589 (42.0) |
| 10 | 66.3 | 66.3 | 33.7 | 33.7 | 12.1 | 96.6 | 0.477 | 503 (35.9) |
| **11** | **59.8** | **74.9** | **25.1** | **40.2** | **14.3** | **96.4** | **0.474** | **384 (27.4)** |
| 12 | 54.3 | 80.5 | 19.5 | 45.7 | 16.4 | 96.2 | 0.497 | 305 (21.7) |
| 13 | 48.9 | 85.4 | 14.6 | 51.1 | 19.1 | 96.0 | 0.531 | 236 (16.8) |
| **Dysglycaemia** | | | | | | | | |
| **FINDRISC-BI** | | | | | | | | |
| 6 | 79.3 | 49.9 | 50.1 | 20.7 | 40.9 | 84.7 | 0.542 | 827 (58.9) |
| 7 | 71.8 | 60.0 | 40.0 | 28.2 | 43.9 | 83.0 | 0.489 | 697 (49.7) |
| **8** | **66.4** | **67.0** | **33.0** | **33.6** | **46.8** | **82.1** | **0.471** | **605 (43.1)** |
| 9 | 54.9 | 74.1 | 25.9 | 45.1 | 48.0 | 79.0 | 0.520 | 487 (34.7) |
| 10 | 49.1 | 81.5 | 18.5 | 50.9 | 53.6 | 78.6 | 0.542 | 390 (27.8) |
| **Modified FINDRISC-BI** | | | | | | | | |
| 7 | 78.6 | 52.0 | 48.0 | 21.4 | 41.7 | 84.8 | 0.526 | 804 (57.3) |
| 8 | 73.5 | 58.4 | 41.6 | 26.5 | 43.5 | 83.5 | 0.493 | 719 (51.2) |
| **9** | **63.8** | **67.6** | **32.4** | **36.2** | **46.2** | **81.1** | **0.486** | **589 (42.0)** |
| 10 | 57.7 | 73.7 | 26.3 | 42.3 | 48.9 | 80.0 | 0.498 | 503 (35.9) |
| 11 | 46.9 | 81.2 | 18.8 | 53.1 | 52.1 | 77.8 | 0.563 | 384 (27.4) |

FINRISC-BI: FINDRISC Bahasa Indonesia; T2DM: type 2 diabetes mellitus; Sen: Sensitivity; FN: False negative; Spe: Specificity; PPV: Positive predictive value; FP: False positive; NPV: Negative predictive value

*Proportion of participants with FINDRISC score above the cut-off value
